# Non-vesicular Lipid Transport Machinery in *Entamoeba histolytica*

**DOI:** 10.3389/fcimb.2018.00315

**Published:** 2018-09-19

**Authors:** Koushik Das, Tomoyoshi Nozaki

**Affiliations:** Graduate School of Medicine, The University of Tokyo, Bunkyō, Japan

**Keywords:** lipid, *E. histolytica*, signaling, protozoan parasites, pathogenesis

## Abstract

Eukaryotic cells are organized into separate membrane-bound compartments that have specialized biochemical signature and function. Maintenance and regulation of distinct identity of each compartment is governed by the uneven distribution and intra-cellular movement of two essential biomolecules, lipids, and proteins. Non-vesicular lipid transport mediated by lipid transfer proteins plays a pivotal role in intra-cellular lipid trafficking and homeostasis whereas vesicular transport plays a central role in protein trafficking. Comparative study of lipid transport machinery in protist helps to better understand the pathogenesis and parasitism, and provides insight into eukaryotic evolution. Amebiasis, which is caused by *Entamoeba histolytica*, is one of the major enteric infections in humans, resulting in 40–100 thousand deaths annually. This protist has undergone remarkable alterations in the content and function of its sub-cellular compartments as well represented by its unique diversification of mitochondrion-related organelle, mitosome. We conducted domain-based search on AmoebaDB coupled with bioinformatics analyses and identified 22 potential lipid transfer protein homologs in *E. histolytica*, which are grouped into several sub-classes. Such *in silico* analyses have demonstrated the existence of well-organized lipid transport machinery in this parasite. We summarized and discussed the conservation and unique features of the whole repertoire of lipid transport proteins in *E. histolytica*.

## Introduction

Eukaryotic cells are organized into distinct membrane-enclosed organelles or compartments. Each organelle has distinctive lipid and protein signature and dedicated function (Holthuis et al., 2003; Lev, 2010). Each cell organelle also assures proper segregation of complex cellular processes catalyzed by metabolic enzymes, structural and regulatory proteins (Lev, 2010). Proteins are precisely distributed to different cell organelles either by their intrinsic signal peptides or through post-translational modifications (Lev, 2010). In contrast, lipids do not have any such signal sequence that determine their accurate intracellular distribution (Lev, 2010). Nevertheless, each organelle differs in its lipid composition (Voelker, 1991; Sprong et al., 2001; Lev, 2010). In general, ER is the main site for lipid synthesis (Blom et al., 2011) and lipids are then transported from ER to site of function. Previous studies suggest that both vesicular and non-vesicular transport machinery are responsible for the delivery of lipids to their final destinations (Lev, 2010). Vesicular transport has an important role in protein trafficking, endocytic and exocytic (secretory) pathways (Lev, 2010). It is an energy dependent process and involves cytoskeletal reorganization (Lev, 2010). However, significant amount of lipids can be transferred by vesicular transport as lipids are the major component of transport vesicles (Lev, 2010). Nonetheless, lipid transport was still identified when vesicular transport was impaired by either depletion of ATP, reduced temperature, or treatment with pharmacological inhibitors (such as brefeldin A and colchicine) (Kaplan and Simoni, 1985; Vance et al., 1991; Li et al., 2014). Lipid transportation was also detected among cell organelles, those are not linked by vesicular transport machinery (e.g., ER/mitochondria and ER/peroxisomes) (Levine, 2004; Holthuis and Levine, 2005). These observations suggest that non-vesicular transport mechanisms have a significant role in intracellular lipid trafficking. Non-vesicular lipid transport in and between organellar membranes is mostly facilitated by three possible methods: lateral diffusion, trans-bilayer flip-flop, and monomeric lipid exchange (Sleight, 1987; Van Meer, 1989; Lev, 2010). Lateral diffusion is responsible for the lateral movement of lipid in a membrane bilayer (Lev, 2010). Although lateral diffusion mostly transport lipid within membranes, this process was also identified between membranes which are linked via membrane bridges (Lev, 2010). Lipids are moved between two layers of the membrane bilayer by the process called trans-bilayer flip-flop (Lev, 2010). This type of movement takes place either spontaneously or mediates by flippases and translocases (Sprong et al., 2001; Lev, 2010). Trans-bilayer flip-flop do not participate directly in inter-organelle lipid transport (Lev, 2010). It can either encourage non-vesicular lipid transport by monomeric lipid exchange or influence vesicular transport through the alteration of membrane curvature, vesicle budding and fusion (Sprong et al., 2001; Lev, 2006, 2010). Monomeric lipid exchange, the primary mechanism of intra-cellular lipid transport is an energy-independent process (Lev, 2010). In this process, lipid monomer is transported from a donor membrane to an acceptor membrane through the cytosol either spontaneously or facilitated by lipid transfer proteins (LTPs) (Lev, 2010). Spontaneous transport of lipid molecule from donor to the acceptor membrane through cytosol is a time- consuming process and insufficient for substantial transport of major lipids (Jones and Thompson, 1989; Mesmin and Maxfield, 2009; Lev, 2010, 2012).

Non-vesicular lipid transport between cellular membranes are greatly facilitated by LTPs, which are the key contributor of organelle-specific lipid distribution and cellular lipid homeostasis (Helle et al., 2013). LTP-mediated lipid transport locally modulates the lipid composition of membranes and consequently regulates various cellular processes including vesicular trafficking, lipid metabolism, and signal transduction (Ohashi et al., 1995; Kim et al., 2015). Furthermore, LTPs can also act as membrane contact sites (MCSs) between the endoplasmic reticulum (ER) and virtually all other organelles, and are involved in the transport of Ca^2+^, metabolites, and lipids (Lev, 2010; Helle et al., 2013).

Amebiasis is caused by a protozoan parasite *Entamoeba histolytica* and one of the major enteric infections in humans. An estimated 50 million people are infected with *E*. *histolytica* worldwide, resulting in 40–100 thousand human deaths annually (Haque et al., 2003; Stanley, 2003; Hung et al., 2012). *In-vivo* growth and survival of *E. histolytica* as an enteric parasite depends on its efficient cellular processes to thrive in adverse host environment (Mittal et al., 2008; Nakada-Tsukui et al., 2009; Vicente et al., 2009). The parasite has two inter-changeable stages consisting of the infective dormant cyst and the proliferative motile trophozoite stages in the life cycle. Inter- conversion between cysts and trophozoites involves complex cellular metabolic processes and essential for transmission of the disease. Lipid and its metabolism apparently play important roles in stage conversion (De Cádiz et al., 2013). Furthermore, this anaerobic or microaerophilic parasite has to overcome a wide variety of environmental and host-derived oxidative and nitrosative stresses during its life cycle (Vicente et al., 2009; Baumel-Alterzon and Ankri, 2014; Pineda and Perdomo, 2017). The pathogenic behavior of this parasite depends on their ability to uptake host nutrients through ingestion and degradation of mammalian cells and tissues. *E. histolytica* trophozoites engulf live and dead host cells through trogocytosis and phagocytosis, respectively (Nakada-Tsukui et al., 2009; Ralston et al., 2014). Contact dependent cytolysis of host cells depends on the secretion of cytolytic proteins, including cysteine proteases (CP) (Que and Reed, 2000) and pore forming peptides (Zhang et al., 2004). Such contact dependent killing and uptake of host cells are likely primed with a receptor-ligand interaction on the surface, which leads to activation of lipid signaling cascades to downstream effectors (Nakada-Tsukui et al., 2009; Somlata et al., 2017).

Lipids can either directly affect the membrane charge and the curvature by their altered local distribution or act as precursors to accelerate the local lipid synthesis by lipid metabolizing enzymes. Furthermore, it has also been established that in the nucleus, distribution and metabolism of lipids and lipid-mediated signaling play essential roles in cell proliferation, differentiation, and stress adaptation in higher eukaryotes (Shah et al., 2013). By analogy, non-vesicular lipid transport mediated by LTPs are likely indispensable for *E. histolytica*. As *E. histolytica* resides in the anaerobic or microaerophilic environment, its mitochondrial functions have highly diverged, resulting in the atypical mitochondrion-related organelles (MROs), called mitosomes, which have drastically modified morphological, structural, and functional features compared to those of the aerobic mitochondria (Makiuchi and Nozaki, 2014). Likewise, *E. histolytica* also lacks well-defined morphologically discernible ER and Golgi apparatus (Perdomo et al., 2016). Lipid transport machinery between such highly divergent organelles in *E. histolytica* could be significantly different from those in the well characterized higher eukaryotes. Thus, investigation on the LTP-mediated lipid transfer in *E. histolytica* should certainly help us to better understand conservation and diversity of this important biological process. Here, we conducted an InterPro domain search (PFAM) analysis on AmoebaDB in order to identify the potential LTP candidates in *E. histolytica*. We also discussed the conservation and unique features of LTPs, and their possible mechanisms and roles in biology and pathophysiology of amebiasis.

## General background of lipid transport by lipid transfer proteins (LTPs)

### Domains of LTPs

**Figure 1:**
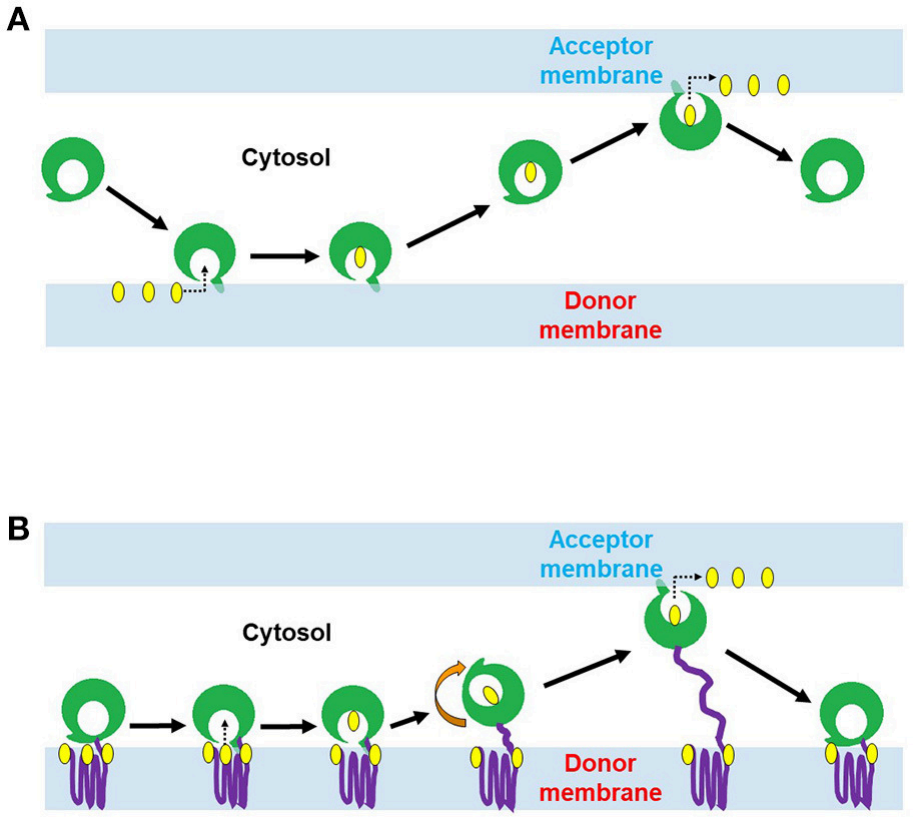
Two proposed mechanisms of lipid transport by lipid transfer proteins (LTPs). **(A)** Lipid conveyance across the cytoplasm by cytosolic LTP. Cytosolic LTP (e.g., STARD containing proteins 1-7) contains only lipid-transfer domain (LTD) (green) but lacks any membrane binding domain. A ligand-free form of LTP interacts with the donor membrane to acquire a lipid molecule (yellow). This interaction induces a conformational change of LTP and opens its lipid binding pocket. Once the lipid molecule (yellow) has occupied the pocket, the protein conformational change occurs again, leading to a lid closure. The ligand-bound form of LTP is targeted to the acceptor membrane to deliver the lipid. **(B)** Lipid transport at MCSs by membrane anchored LTP (e.g., ceramide transfer protein, CERT; oxysterol-binding protein, ORP3). LTP anchors with the donor membrane via its membrane binding domain (purple) and exposes its LTD (green) to the cytoplasm. LTD can extract the lipid molecule (yellow) from the donor membrane and deliver it to a closely positioned acceptor membrane.

Non-vesicular lipid transport is catalyzed by LTPs. LTPs can extract lipids from the donor membrane and deliver them to the acceptor membrane. This type of transfer involves a special lipid-transfer domain (LTD) that can form a lipid binding cavity, which binds and accommodate the hydrophobic moieties of lipid molecules from the aqueous environment (Helle et al., 2013). A combination of hydrogen bonds and hydrophobic interactions secure the lipid binding and influence the binding affinity (Lev, 2010). In addition to LTDs, LTPs often possess different combinations of membrane/organelle targeting domains, which direct them to special cellular compartments (Helle et al., 2013), such as pleckstrin homology (PH), protein kinase C (PKC) conserved 1 (C1) and PKC conserved 2 (C2) domains. C1 domain (~50 amino acids, a cysteine-rich compact structure) was first identified in protein kinase C (PKC) as the binding site for di-acyl-glycerol (DAG) and phorbol ester (Cho, 2001). The binding site has cationic residues, which accelerate the Ca^+2^ dependent recruitment and adsorption of the C1 domain to the anionic membrane surfaces (Cho, 2001), while the hydrophobic tip of the domain penetrates the membrane to bind with DAG that is partially buried in the membrane (Cho, 2001). C2 domain (~130 amino acids, Ca^+2^ binding site) was found in PKC, cytosolic phospholipase A_2_ (PLA_2_), phospholipase C (PLC), phospholipase D (PLD) and phosphoinositide (PtdIns phosphate, PI) 3-Kinase (Cho, 2001). Ca^+2^ ions assist the membrane targeting of C2 domain either through providing a bridge between the C2 domain and anionic phospholipids or induce intra-domain conformational change which in turn triggers membrane protein interactions (Cho, 2001). Sub-cellular localization of C2 domain depends on its phospholipid specificities. C2 domain of PKC prefers anionic phospholipids rapidly translocate to PM, while the C2 domain of PLA_2_ is selective to phosphatidylcholine (PC) localized to the perinuclear region in response to Ca^+2^ import (Cho, 2001). LTPs are broadly classified into two major classes based on their domain architecture: (i) cytosolic LTPs, which lack any membrane binding domain, and (ii) membrane anchored LTPs, which contain some membrane binding domain(s) and function to form a MCS. Cytosolic LTPs could possibly facilitate intra-cellular transport of lipid through a sequential process involving interaction of the LTP with donor membrane (Lev, 2012) followed by the opening of hydrophobic cavity, lipid extraction and dissociation of LTP from donor membrane, movement through cytosol in a “closed” transport competent conformation (Kasper and Helmkamp, 1981; Helmkamp, 1986; Nichols, 1988; Rueckert and Schmidt, 1990; Wirtz, 1991; Gadella and Wirtz, 1994; Wirtz et al., 2005; Lev, 2012). The transport is completed by the interaction of LTP with an acceptor membrane, opening of the lipid binding cavity, and desorption of lipid molecule (Lev, 2012) (Figure 1A). In contrast to cytosolic LTPs, LTPs with two targeting domains/motifs for two different organelle membranes are naturally directed to membrane contact sites (MCSs) between these two cellular compartments (Lev, 2010; Helle et al., 2013). Such MCSs bring two membranes from different organelles in a close vicinity (at 10-20 nm), which favor lipid exchange between such closely apposed membranes (Figure 1B) and also regulate intra-cellular Ca^+2^ and signaling processes (Levine, 2004; Voelker, 2005; Levine and Loewen, 2006; Giorgi et al., 2009; Lebiedzinska et al., 2009; Lev, 2010).

### Biological roles of LTPs

LTPs have different modes of action: First, LTPs can facilitate vectorial and often bidirectional lipid transfer as shown in intact living cells and *in vitro* (Helle et al., 2013). Second, LTPs have been recognized as lipid presenting proteins for lipid metabolizing enzymes (Kular et al., 1997, 2002; Cockcroft and Garner, 2013). It can transiently modulates the intracellular lipid metabolism by providing lipid substrates to lipid metabolizing enzymes during signal transduction pathways under various physiological and cellular conditions (Cockcroft and Garner, 2013; Fayngerts et al., 2014). Third, LTPs can act as lipid sensors by altering their affinity to other associated proteins in response to the binding to lipids or bio-membranes (Lev, 2010). Fourth, LTPs can facilitate transient alterations in lipid distribution of a bio-membrane by extracting or delivering of lipid molecules to a certain region of the membrane, or through changing the lipid phase in a particular membrane portion to which it is bound (Lev, 2010). LTP can employ more than one of these mechanisms and regulates the following cellular process (Ohashi et al., 1995; Kim et al., 2015).

#### Intra-cellular lipid trafficking

Inter-organelle lipid transport facilitated by steroidogenic acute regulatory protein-related lipid transfer (START) domain containing proteins is mostly studied. Mammalian START domain containing proteins are sub-divided into broad sub-families based on their domain organization (membrane targeted and soluble START) and lipid specificities (cholesterol/oxysterol binding proteins and phospholipid/ sphingolipid binding proteins). STARD1 sub-family (contain of STARD1 and STARD3/MLN64) and STARD4 sub-family (comprised of STARD4, STARD5, and STARD6) transport cholesterol to various cell organelles (Alpy and Tomasetto, 2005; Clark, 2012). STARD2 sub-family is composed of STARD2, STARD7, STARD10, and STARD11/CERT (Alpy and Tomasetto, 2005; Clark, 2012). STARD2, 7 and 10 transport PC/PE (Alpy and Tomasetto, 2005; Clark, 2012), while STARD11/CERT transport ceramide from ER to Golgi complex (Alpy and Tomasetto, 2005; Clark, 2012; Kumagai et al., 2014). Among the above mentioned START proteins, STARD3/MLN64 and STARD11/CERT have additional membrane targeted domain, rest possess only STARD domain and are cytosolic (Alpy and Tomasetto, 2005; Clark, 2012).

#### Lipid supply for metabolism and signal transduction

The receptor-ligand mediated signaling processes such as trogocytosis, phagocytosis, pinocytosis and exocytosis involves an array of PIs and their metabolizing enzymes (PI 4-kinases, PtdIns4P 5-kinases, PI 3-phosphatases, PI 5-phosphatases, and non-specific phosphatases), residing in various cell organelles (Kölsch et al., 2008; Thomas, 2012; Haastert et al., 2013; Levin et al., 2015). However, PtdIns, the main precursor of PIs is synthesized in the ER and needs to be transported by LTPs (also known as PtdIns transfer proteins, PITPs) to cell organelles for the generation of PIs pools during these cellular processes. PA is produced from diacylglycerol (DAG) by DAG kinases at the plasma membrane (PM), also transported back to the ER by PITPs for replenishment of PtdIns at the ER (Cockcroft and Garner, 2013). This example illustrates that LTPs can function as lipid presenting proteins for lipid metabolizing enzymes and subsequently modulates the lipid metabolism associated with signal transduction pathways under various physiological and cellular conditions.

#### Lipid sensing, and regulation of vesicular trafficking

LTPs can function as lipid sensors and regulate Golgi-mediated vesicular trafficking, exocytosis (Litvak et al., 2005; Peretti et al., 2008; Mattjus, 2009), as well explained for Sec14 in *S. cerevisiae* (Curwin et al., 2009). Sec14 involved in intra-cellular transport of either phosphatidylcholine (PC) or phosphatidylinositol (PtdIns) between the ER and the Golgi complex (Bankaitis et al., 1990; Lev, 2010). However, the PtdIns–PC exchange activity of Sec14 is not involved in the Golgi secretory function. Instead, Sec14 functions as PC sensor, can sense the PC level in Golgi and respond to increased PC level by inhibiting its production from diacylglycerol (DAG) through cytidine diphosphate (CDP)-choline pathway (McGee et al., 1994; Skinner et al., 1995; Lev, 2010). In this way, Sec14 regulates a critical level of DAG and PC in Golgi, which is crucial for Golgi mediated vesicular trafficking, exocytosis and viability of *S. cerevisiae* (Bankaitis et al., 1989; Lev, 2010). Sec14 can function as both as a PC sensor and as a PtdIns-presenting protein, which transmits PC metabolic information to PI synthesis (Schaaf et al., 2008; Lev, 2010). Oxysterol-binding-protein-related proteins (ORPs) interact with Rab GTPases and control intra-cellular movement of transport vesicles as described for ORP1L from higher eukaryotes (Johansson et al., 2007; Rocha et al., 2009; Lev, 2010). ORP1L can induce the formation of the membrane contact site (MCS) between the ER and late endosomes, by undergoing conformational changes in response to lower cholesterol content in late endosomes (Johansson et al., 2007; Rocha et al., 2009; Lev, 2010). Priming and docking of the exocytic vesicle complex with the PM also requires an PITP mediated transient alteration in lipid [e.g., PtdIns(4,5)P2] distribution at the site of exocytosis (Lev, 2010; Thomas, 2012).

#### Modulation of nuclear lipid signaling and associated nuclear functions

A repertoire of lipid (PI, PA, and DAG) metabolizing enzymes, their lipid substrates, byproducts and downstream effectors, involved in various aspects of transcription, chromatin remodeling, mRNA maturation, cell proliferation, differentiation, and stress management (Tanaka et al., 1999; Martelli et al., 2002; Audhya and Scott, 2003; Irvine, 2003; Balla and Balla, 2006; Matsubara et al., 2006; Carman and Henry, 2007; Demmel et al., 2008; Mishkind et al., 2009; Ren et al., 2010; Jang and Min, 2011; Shah et al., 2013; Symeon, 2013; Jülke and Ludwig-Müller, 2015; Karlsson et al., 2016), have been identified in eukaryotic nucleus. In order to maintain the critical level of lipid in the nucleus, the precursor for lipid biosynthetic enzymes needs to be transported to the nucleus by LTPs. Other than function as a lipid exchanger, LTP can interacts with other nuclear associated proteins and regulates the nuclear functions as described for microsomal triglyceride transfer protein, which regulate lipid homeostasis and interacts with RNA helicase DDX3, hepatocyte nuclear factor 4 (HNF4) and small heterodimer partner (SHP) (Tsai et al., 2017). An LTP can also modify nuclear transport via its interaction with a nuclear pore component (Nup62) as described for sterol transporter, ORP8 (Zhou et al., 2011; Béaslas et al., 2012).

#### Cytoskeleton organization, adhesion, and motility

Several LTP homologs in higher eukaryotes were reported to be associated with cytoskeleton organization, cell adhesion, and motility. For instances, ORP3 and its close relative ORP7 interacts with R-Ras and regulates cytoskeleton organization, cell adhesion, migration (Goldfinger et al., 2007; Weber-Boyvat et al., 2013), while STARD8/12/13 (START proteins with Rho GTPase activating protein (Rho-GAP) domain) are also involved in cytoskeleton organization and migration of a cancer cell line (Alpy and Tomasetto, 2005).

## Identification of lipid transfer protein (LTP) homologs in *E. histolytica*

### Identification, domain organization of *E. histolytica* LTP homologs

**Figure 2:**
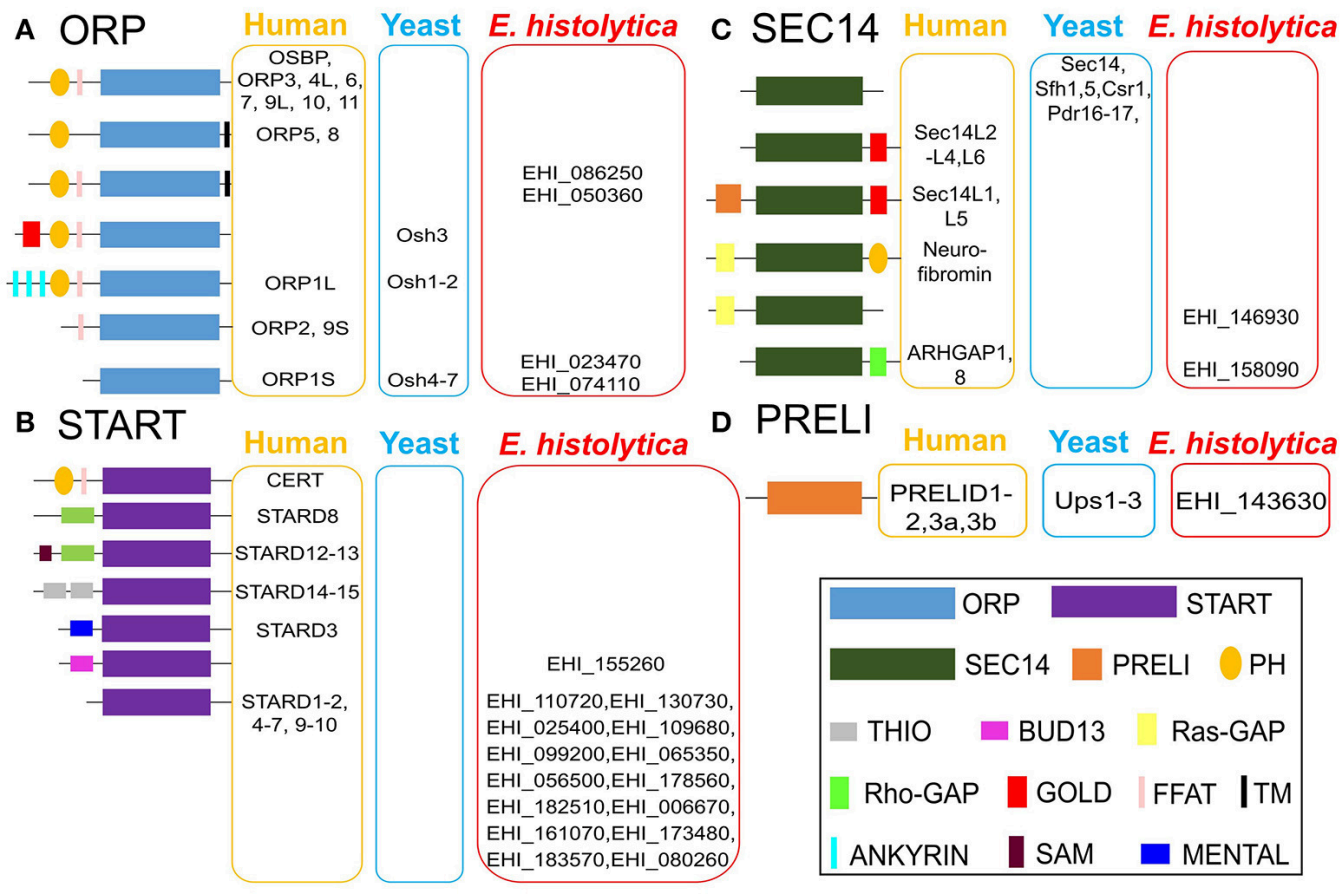
Classification and domain organization of lipid transfer proteins (LTPs) from human, yeast, and *E. histolytica*. Based on domain organization and cellular localization, LTPs are classified into cytosolic and membrane-bound LTPs. Cytosolic LTPs possess only lipid transfer domains (LTDs) such as oxysterol-binding protein (OSBP)-related domain (ORD, bind to sterols and to PtdIns4P), steroidogenic acute regulatory protein (StAR)-related lipid transfer (START) domain (bind to either sterols, phospholipids, or ceramides), Sec14 domain (bind to PC and PtdIns), and protein of relevant evolutionary and lymphoid interest (PRELI domain, binds to PA), all of which can shelter the hydrophobic moieties of various lipid ligands from the aqueous environment. Membrane bound LTPs have various combinations of LTDs with other additional membrane-anchored domains/motifs such as pleckstrin-homology (PH) domain, diphenylalanine-in-an-acidic-tract (FFAT) motif, Golgi dynamics (GOLD) domain, function as membrane contact sites (MCSs). *E. histolytica* genome has 22 potential LTP homologs, **(A)** four OSBP-related protein (ORP), **(B)** 15 START, **(C)** two Sec14, and **(D)** single PRELI candidates. AmoebaDB ID of *E. histolytica* LTP homologs are shown.

We conducted an InterPro domain search (PFAM) analysis on AmoebaDB version 38 (released 5th July, 2018) in order to identify the potential LTP candidates in *E. histolytica* HM-1:IMSS. The *E. histolytica* HM-1:IMSS genome encodes a diverse repertoire of 22 potential lipid transfer protein (LTP) homologs [four oxysterol-binding-protein-related (ORP) proteins, 15 steroidogenic acute regulatory protein-related lipid transfer (START) proteins, two Sec14 like proteins, and one protein of relevant evolutionary and lymphoid interest (PRELI) domain containing protein] (Figure 2) with different E-value (as E-value provided by AmoebaDB). We further verify each of these twenty two potential LTP homologs for possessing of the indicated lipid transfer domain (LTD) [for instances, STARD, ORD, Sec14 and PRELI domains] by NCBI conserved domain search analysis and position of individual domain in each homolog were defined. Twenty two potential LTP homologs were then compared with LTP homologs previously studied in other eukaryotes, in particular, human and *Saccharomyces sp*., and grouped based on their domain organization (Figure 2). Note that information on non-vesicular lipid transport are mostly available in human (Curwin and McMaster, 2008), yeast (Bankaitis et al., 1989, 1990; Im et al., 2005), plant (Li et al., 2016) and little in *Plasmodium sp*. (Van Ooij et al., 2013; Hill et al., 2016)]. Mutual amino acid identities among LTP homologs from *E. histolytica* and their counterparts in human and yeast (as per the classification in Figure 2), calculated with ClustalW, revealed that *E. histolytica* LTPs are significantly divergent from those of human and other eukaryotes (Data not shown in this review). A summary table indicates the repertoire of LTP homologs will be analyzed in *E. histolytica* (Table 1).

**Table 1:** The repertoire of LTP homologs from *E. histolytica* identified and analyzed in this study.

**LTP homolog^a^**	**Accession number^b^**	**Domain organization^c^**
START	EHI_110720, EHI_130730, EHI_025400, EHI_109680, EHI_099200, EHI_065350, EHI_056500, EHI_178560, EHI_182510, EHI_006670, EHI_161070, EHI_173480, EHI_183570, EHI_080260	START^e^
	EHI_155260	BUD13^f^, START
ORP	EHI_023470, EHI_074110	ORP^g^
	EHI_086250, EHI_050360	PH^h^, FFAT^i^, ORP, TM^j^
Sec14	EHI_146930	Ras-GAP^k^, Sec14^l^
	EHI_158090	Sec14, Rho-GAP^m^
PRELI	EHI_143630	PRELI^n^

*Twenty two potential LTP homologs of E. histolytica, their accession numbers and domain structure. Refer to Figure 2 for classification and domain organization*.

a *LTP homolog in E. histolytica as per AmoebaDB database*.

b *AmoebaDB ID*.

c *Domain organization based on NCBI conserved domain search analysis*.

e *steroidogenic acute regulatory protein-related lipid transfer (START) domain*.

f *BUD (BurrH domain) 13 domain*.

g *oxysterol-binding-protein-related (ORP) domain*.

h *pleckstrin-homology (PH) domain*.

i *diphenylalanine-in-an-acidic-tract (FFAT) motif*.

j *Transmembrane (TM) domain*.

k *Ras-GAP domain*.

l *Sec14 domain*.

m *Rho-GAP domain*.

n *protein of relevant evolutionary and lymphoid interest (PRELI) domain*.

#### ORPs

*E. histolytica* possess four ORP homologs (EHI_086250, EHI_050360, EHI_023470, and EHI_074110), which could mediate sterol transport between the ER and other organelles (Figure 2). Among four ORP homologs, two of them (EHI_023470 and EHI_074110) contain only ORP-related domain (ORD) and lacks any potential membrane anchoring domain, which indicates that they are likely localized in the cytosol. The two other ORP homologs (EHI_086250 and EHI_050360) possess the PH domain and the diphenylalanine-in-an-acidic-tract (FFAT) motif in the amino terminus and a single TM domain in the carboxyl terminus (Figure 2). PH domain is known to interact with specific PIs such as PtdIns4P found on the Golgi membrane and the PM (Helle et al., 2013), while FFAT is known to bind to ER proteins (Helle et al., 2013). Thus, these two TM domain-containing ORP candidates likely act to form a potential MCS between the ER and the Golgi and/or the ER and the PM. They potentially bring these two organelle membranes close enough to facilitate lipid transport as observed in OSBP-mediated sterol transport (Raychaudhuri et al., 2006; Schulz and Prinz, 2007; Raychaudhuri and Prinz, 2010; Olkkonen, 2015) and CERT-mediated ceramide transport at the ER–Golgi MCSs in mammals (Hanada, 2010; Lev, 2010; Kumagai et al., 2014).

#### START domain containing proteins

Among 15 START domain containing proteins, most of them (except EHI_155260) contain only START domain, which likely indicate their cytosolic localization (Figure 2). Only one protein (EHI_155260) has an additional BUD13 domain (Figure 2), which was previously reported to have a role in mRNA splicing and retention in higher eukaryotes (Scherrer and Spingola, 2006). EHI_155260 also has the potential NLS and NES, which indicates that it can potentially relocate between the nucleus and the cytoplasm, depending upon physiological and environmental conditions. This domain organization of EHI_155260 is unique to *E. histolytica*. Low mutual similarity among *E. histolytica* START protein homologs (Data not shown in this review) indicate their diverse ligand specificities and functions, as observed in higher eukaryotes (Alpy and Tomasetto, 2005).

#### Sec14s

*E. histolytica* has two potential Sec14 homologs (EHI_146930 and EHI_158090), which also possess Ras GTPase activating protein (Ras-GAP) or Rho-GAP, respectively (Figure 2). Sec14 protein was first identified in budding yeast, essential for the transport of secretory proteins from the Golgi complex (Mousley et al., 2006; Sirokmány et al., 2006; Curwin et al., 2009). Sec14 homologs possessing Ras- GAP or Rho-GAP domain has also been identified in human (Curwin and McMaster, 2008). p50Rho-GAP/ARH-GAP1 (Sec14 homolog with Rho-GAP domain) (Figure 2) is present on the endosomal membrane, where it co-localizes with internalized transferrin receptor (Sirokmány et al., 2006). The Sec14 domain of p50Rho-GAP is essential for its endosomal targeting. p50Rho-GAP forms *in vivo* a complex with Rab5 and Rab11 on endosomal membranes through its Sec14 domain. Thus, Sec14 domain, which was previously known as a phospholipid binding module, mediates protein-protein interactions with Rab and Rho-GTPases and regulates receptor-mediated endocytosis (Sirokmány et al., 2006). *E. histolytica* Sec14 homolog with Rho-GAP domain (EHI_158090) could potentially play a similar function in endocytic and trogo/phagocytic processes.

#### PRELI domain containing proteins

The *E. histolytica* genome contains a single PRELI-like domain containing protein (EHI_143630) (Figure 2), which may be involved in lipid homeostasis of its highly divergent mitochondrion-related organelle, as previously described for aerobic mitochondria from higher eukaryotes (Miliara et al., 2015; Tatsuta and Langer, 2016). *E. histolytica* possesses a highly divergent form of the mitochondrion called mitosome, which is unique in its content and function to *Entamoeba* (Makiuchi and Nozaki, 2014). It is mainly involved in sulfate activation, and important for parasite growth and differentiation (Mi-ichi et al., 2009, 2015). Since, *E. histolytica* do not possess any canonical mitochondria (Makiuchi and Nozaki, 2014), it will be interesting to study whether function of PRELI homolog in *E. histolytica* (EHI_143630) is evolutionary conserved or it has a distinct cellular function unique to this protozoan parasite.

#### Other proteins known to be involved in lipid transfer in other organisms, but missing in *E. histolytica*

*E. histolytica* lacks some of LTP homologs known to be present and functional in other eukaryotes (Figure 2). *E. histolytica* has no homologs for human PITPs, similar as *Saccharomyces sp*. (Nile et al., 2010). The *Saccharomyces* genomes contain several Sec14 homologs that function as PITPs (Phillips et al., 2006). *E. histolytica* possesses a panel of START domain protein homologs, some of which could potentially function as PITP as reported in *Plasmodium falciparum* (Van Ooij et al., 2013; Hill et al., 2016). *E. histolytica* also lacks a homolog of eukaryotic synaptotagmin-like, mitochondrial and PH domain (SMP) containing proteins (Helle et al., 2013). In yeast SMP-containing homologs (Nvj2, Tcb1, Tcb2 and Tcb3) are localized at various MCSs, which indicates their common function at MCSs (Helle et al., 2013). Moreover, the ER–mitochondria encounter structure (ERMES) components (the ER protein, Mmm1, the cytosolic protein, Mdm12, and the OMM protein, Mdm34) also possess SMP domain (Helle et al., 2013). Since the *E. histolytica* genome encodes none of ERMES components and other SMP homologs (Figure 2), it is plausible that some LTP homologs could potentially function at the MCSs. It is known in other organisms that some LTP homologs with unique domain organization are unique to a particular organism. For instance, Osh3 (of ORP sub-class), Sec14, Pdr16, Pdr 17, Sfh1 and Sfh5 (of Sec14 sub-class) are unique to yeast (Figure 2). Similarly, EHI_155260 (of START sub-class), EHI_086250 and EHI_050360 (of ORP sub-class) are unique to *E. histolytica* (Figure 2).

### mRNA expression of LTP homologs in *E. histolytica* HM-1:IMSS

**Figure 3:**
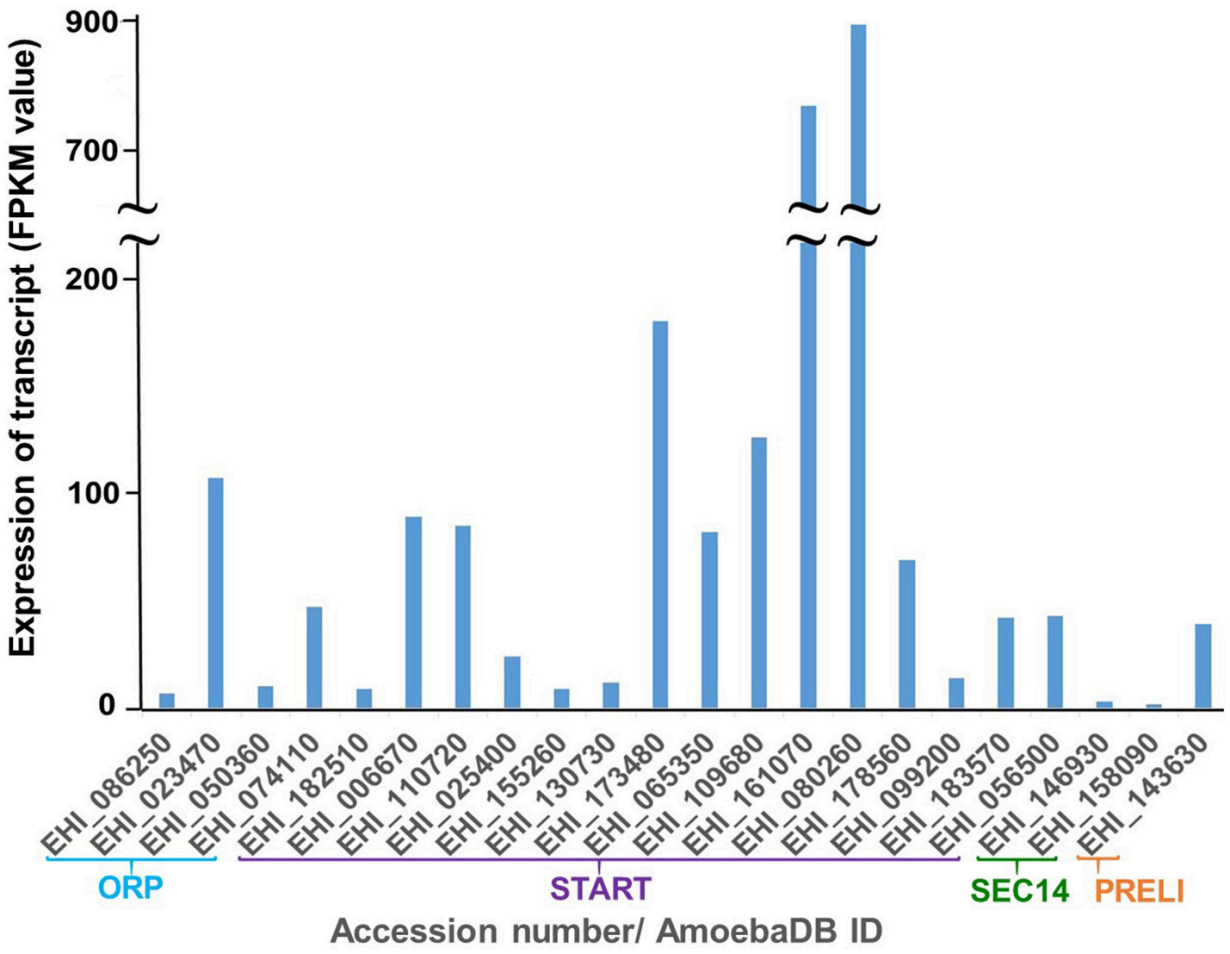
Relative mRNA expression of LTP homologs in *E. histolytica* HM-1:IMSS. Survey of the *E. histolytica* genome has identified a total of 22 LTP homologs. The levels of mRNA expression are shown with FPKM (fragments per kilobase of exon per million fragments mapped) value as per AmoebaDB. Three members of *E. histolytica* START protein homologs (EHI_080260 followed by EHI_161070 and EHI_173480) show the highest mRNA expression in HM-1:IMSS strain among all LTP candidates. Among 4 ORP candidates, EHI_023470, and EHI_074110, both of which contain only ORD and are possibly cytosolic (see Figure 2 for their classification and domain organization), show higher levels of expression compared to the two remaining membrane-bound LTP homologs (EHI_086250 and EHI_050360). Sec14 homologs (EHI_146930 and EHI_158090) show low expression levels among all identified LTP candidates.

Relative steady-state levels of mRNA expression of a panel of 22 potential LTP homologs from *E. histolytica* (15 START proteins, 4 ORPs, 2 Sec14s and 1 PRELI domain proteins) were investigated using data available at AmoebaDB (Hon et al., 2013). Three members of *E. histolytica* START protein homologs showed higher mRNA expression in HM-1:IMSS compared to other LTP candidates (in a descending order of EHI_080260, EHI_161070, and EHI_173480) (Figure 3). Three START protein homologs (EHI_182510, EHI_155260 and EHI_130730) showed very low levels of expression (Figure 3). Interestingly, the reptilian sibling *Entamoeba* species, *E. invadens*, has two EHI_155260 homologs (EIN_257190, EIN_107840). Similar patterns of upregulation of these two gene transcripts were observed during encystation (data not shown) (De Cádiz et al., 2013), which indicates their potential roles in cell differentiation, as previously shown for LTP during somatic embryogenesis in *Arabidopsis thaliana* (Potocka et al., 2012). Among 4 ORP candidates, EHI_023470 and EHI_074110 are more highly expressed than EHI_086250 and EHI_050360 (Figure 3). Two Sec14 homologs (EHI_146930 and EHI_158090) showed relatively low expression levels among all identified LTP candidates (Figure 3).

## Biological significance of lipid transfer in *E. histolytica*

### Previous reports on lipids and their trafficking in *E. histolytica*

The structure and PM components of *E. histolytica* were studied previously (Espinosa-Cantellano and Martínez-Palomo, 1991), which has identified several surface antigens, adherence proteins, amoebic enzymes (for instances, collagenase, phospholipase A, neuraminidase, cysteine proteases) and fibronectin receptor. PM of *E. histolytica*, enriched in cholesterol, phosphatidylethanolamine (PE), ceramide aminoethyl phosphonate (CAEP) was also reported (Espinosa-Cantellano and Martínez-Palomo, 1991). Previous studies on lipids in *E. histolytica* mostly suggested their roles in parasite growth, proliferation, differentiation, and virulence. It was shown that PC-cholesterol liposomes enabled *in vitro* cultured trophozoites to retain their virulence-associated biological functions such as endocytosis, erythrophagocytosis, expression of surface molecules, protease activity, and liver abscess formation in hamsters. This indicates the contribution of lipids to parasite virulence (Serrano-Luna et al., 2010). Both the unique phospholipid compositions and the high cholesterol content in the amoebic membranes were shown to protect the parasite from self-destruction by its own pore-forming toxins (Andrä et al., 2004). Castellanos-Castro et al. has recently shown that lysobisphosphatidic acid is generally involved in endocytosis (i.e., pinocytosis and erythrophagocytosis). Lysobisphosphatidic acid was demonstrated to be localized in Rab7A positive vesicles in quiescent (non-phagoctytic) conditions and during a late phase of erythrophagocytosis (Castellanos-Castro et al., 2015). It was shown that lipopeptidophosphoglycan (EhLPPG) is also involved in adherence of *E. histolytica* trophozoites to intestinal epithelial cells, similar to other adhesive molecules like, Gal/GalNAc lectin, serine rich *E. histolytica* proteins (SREHP), and lysine glutamic acid rich protein 1 (KERP1) (Stanley et al., 1990; Dodson et al., 1999; Lauwaet et al., 2004; Seigneur et al., 2005). EhLPPG is also recognized by both the innate and the adaptive immune systems and stimulates cytokine production from human monocytes, macrophages, and dendritic cells (Wong-Baeza et al., 2010). EhLPPG induces *in vitro* formation of human neutrophil extracellular traps (NETs) (Ávila et al., 2016). Sulfolipids are one of the terminally synthesized bio-molecules of sulfur metabolism, shown to play an important role in trophozoites proliferation and differentiation processes (Mi-Ichi et al., 2017). For instance, fatty alcohol disulfates plays a crucial role in trophozoites proliferation (Mi-Ichi et al., 2017). Cholesteryl sulfate, another sulfolipid plays a central role in encystation, a differentiation process from the motile trophozoites to the dormant cysts (Mi-Ichi et al., 2017). Furthermore, sulfur metabolism, in which sulfolipids are generated, is not conserved in other free-living amoebae, indicating a causal relationship of sulfur metabolism with parasitism (Mi-Ichi et al., 2017). Lysophosphatidylinositol was shown to stimulate natural killer T (NKT) cells and to induce selective production of IFN-γ but not IL-4 in a CD1-d restricted manner in murine systems (Aiba et al., 2016). The localization of PI (3,4,5)-trisphosphate [PI(3,4,5)P3 or PIP(3)] in *E. histolytica* during various endocytic processes was studied using glutathione S-transferase (GST)- and green fluorescent protein (GFP)-labeled PH domains as lipid biosensors (Byekova et al., 2010). PIP(3) specific biosensor was accumulated at extending pseudopods and also localized in the phagocytic cup during erythrophagocytosis. However, no such localization of the biosensor was observed in pinocytic compartment during pinocytosis. *E. histolytica* maintains a high steady state level of PIP(3) in its PM irrespective of serum concentration (Byekova et al., 2010).

However, non-vesicular lipid transport machinery and the function of LTPs remained largely unexplored in *E. histolytica*. There are two previous reports where the lipid trafficking in *E. histolytica* was described (Pina-Vázquez et al., 2014; Bolaños et al., 2016). Piña- Vázquez et al. identified a START domain containing protein in *E. histolytica* (EHI_110720, also present in our list of *E. histolytica* LTP homologs) and named it as *E. histolytica* phosphatidylcholine transfer protein-like (EhPCTP-L). They identified EhPCTP-L by virtue of interaction with anti-chicken embryo caveolin-1 monoclonal antibody, which indicates their potential role in caveola-mediated endocytosis. EhPCTP-L mainly binds to anionic phospholipids phosphatidylserine (PS) and PA, and is localized to the PM and the cytosol (Pina-Vázquez et al., 2014). However, the essential biochemical characteristics as LTPs, i.e., lipid transport activity, was not reported in this study. START protein homolog (EHI_178560) of *E. histolytica* is essential for parasite growth as observed in the previous study by Solis et al. Double stranded RNA (dsRNA) mediated silencing of EHI_178560 causes growth retardation of *E. histolytica* trophozoites (Solis et al., 2009). Mfotie et al. also reported that plant derivatives that showed anti-amoebic activity also caused the repression of gene expression of another START homolog (EHI_161070) of *E. histolytica* (Mfotie Njoya et al., 2014). Bolaños et al. recently reported that *E. histolytica* NPC1 (EhNPC1) and EhNPC2 proteins responsible for the trafficking of exogenous cholesterol in *E. histolytica* trophozoites and also influence the phagocytosis process (Bolaños et al., 2016). However, these previous studies only partially characterized the roles of LTPs in the trafficking of ingested lipids in *Entamoeba*. The complex network of intra-cellular lipid transport machinery mediated by diverse LTP homologs in *E. histolytica* remains elusive.

### Predicted roles of LTPs in *E. histolytica*

#### Role of *E. histolytica* LTPs in phagocytosis, trogocytosis, endocytosis, signal transduction, and lipid presentation

*E. histolytica* is highly capable of engulfment of the host cells and microorganisms by two distinct processes. In phagocytosis, the parasite engulfs the dead host cells and bacteria as whole, while in trogocytosis the parasite nibbles parts of the live host cells (Nakada-Tsukui et al., 2009; Ralston et al., 2014; Somlata et al., 2017). These two distinct cellular processes are initiated by being triggered by different ligands (on dead/live host cells and microorganisms), and likely activate similar but different cascades of events (Somlata et al., 2017). It is possible that different LTPs may be selectively involved in phagocytosis and trogocytosis. Similarly, *Entamoeba* also depends on receptor-mediated endocytosis/pinocytosis for the transport of nutrients from the extracellular environment (Avalos-Padilla et al., 2015). Such receptor-ligand mediated signaling process is often initiated by PIs (Nakada-Tsukui et al., 2009; Somlata et al., 2017).

*E. histolytica* possess several classes of PI metabolizing enzymes such as: PtdIns 4-kinase (EHI_148700) and PtdIns4P 5-kinases (EHI_153770, EHI_049480), and a panel of PI phosphatases (14 isotypes of PI 3-phosphatase, 6 isotypes of PI 5- phosphatase, and 3 isotypes of non-specific PI phosphatase). Identification of a repertoire of PI kinases and phosphatases enforces the notion that *E. histolytica* LTPs also participate in the constant replenishment of PIs at all cellular compartments at various physiological conditions. *E. histolytica* also possesses 5 putative DAG kinases (data not shown), thus is able to produce PA from DAG. This is consistent with the hypothesis that the reciprocal transport of PtdIns and PA between the ER and the PM (Cockcroft and Garner, 2013) occurs and is mediated by some members of LTPs as lipid presenting proteins.

#### Role of *E. histolytica* LTPs in the secretion of hydrolytic enzymes and regulation of vesicular trafficking

Trafficking and secretion of lysosomal hyodrolases such as CPs contributes to both cytolysis of host tissues and degradation of internalized host cells and microorganisms (Nakada-Tsukui et al., 2005; Mitra et al., 2007). Thus, vesicular trafficking that regulates intracellular CP transport plays a pivotal role in virulence and parasitism of *E. histolytica*. CP trafficking and secretion are regulated via cysteine protease binding family protein (CPBF) 1 (Furukawa et al., 2012; Nakada-Tsukui et al., 2012; Marumo et al., 2014), Rab GTPases (Saito-Nakano et al., 2004, 2005; Mitra et al., 2007; Hanadate et al., 2016), the retromer-like complex (Nakada-Tsukui et al., 2005), and intrinsic CP inhibitors (Sato et al., 2006), and also presumably by priming and docking of CP-containing vesicles with the exo-cyst complex that tethers at the site of exocytosis on the PM (Nakada-Tsukui et al., 2005). As LTPs can function as lipid sensors to regulate Golgi-mediated (or post-Golgi) vesicular trafficking, as above explained for Sec14, it is possible that two Sec14 homologs (EHI_146930 and EHI_158090) that also possess either Rho- GAP or Ras-GAP domain (Figure 2) can regulate the Golgi-mediated secretory function, in a similar mechanism as described previously (Curwin et al., 2009). The four ORP homologs (Figure 2) may interact with Rab GTPase and control trafficking of the transport vesicles as described for ORP1L in higher eukaryotes (Johansson et al., 2007).

#### Role of *E. histolytica* LTPs in nuclear lipid transport and signaling

The nucleus plays indispensable roles in cell proliferation, differentiation, and stress management (Shah et al., 2013). Stage conversion between the two forms in the life cycle requires remarkable alterations in cellular components, metabolism, transcriptional, and post- transcriptional/translational regulations of gene expression, and involves a complex and dynamic signaling events induced by extracellular stimuli (Mittal et al., 2008; Vicente et al., 2009; De Cádiz et al., 2013). Moreover, *E. histolytica* is also harassed by a wide variety of environmental and host-derived stresses, such as fluctuation in glucose concentrations, changes in pH, pO2, temperature, and attack by oxidative and nitrosative stresses from neutrophils and macrophages (Husain et al, 2010; Husain et al., 2012; Nagaraja and Ankri, 2018)*. E. histolytica* senses extracellular stress and accordingly makes necessary amendment in its physiology and metabolism for survival and transmission.

Although the lipid transport, metabolism and signaling in the nucleus and the roles of LTPs in nuclear associated functions are largely unknown, it is conceivable that one START domain containing protein (EHI_155260), which possess BUD13 domain and potential NLS and NES, is involved in nuclear lipid transport, more specifically shuttling between the nucleus and the cytoplasm, and signaling, and thus may be important for growth, stage conversion, and/or evasion from various stresses. In addition, some of the PI and PA metabolizing enzymes in *E. histolytica* (data not shown here) contain putative NLS and NES, indicating their potential roles in nuclear lipid homeostasis by nuclear-cytoplasmic shuttling, as well described in higher eukaryotes (Davis et al., 2015). The *E. histolytica* genome encodes a few PI-binding downstream effectors including a plant homeodomain (PHD) finger-containing protein (EHI_138970), which also contains NLS. Thus, it is conceivable that a panel of these proteins coordinately function in the nucleus. As mentioned above (3.2), *E. invadens* apparently possesses two homologs (EIN_257190, EIN_107840) of the BUD13/NLS/NES-containing START domain containing protein (a single protein in *E. histolytica*, EHI_155260). These *E. invadens* genes showed upregulation of gene expression during encystation (De Cádiz et al., 2013), which indicates their potential role in the nucleus during differentiation, as observed in other eukaryotes (Potocka et al., 2012).

#### Role of *E. histolytica* LTPs in motility, adherence, and cytoskeletal reorganization

It is conceivable that two SEC14 homologs containing Rho-GAP and Ras-GAP (EHI_146930 and EHI_158090, respectively) are involved in cytoskeletal reorganization associated with cell motility and adherence to the host cells and microorganisms. The genome of *E. histolytica* also contains four ORP (EHI_086250, EHI_050360, EHI_023470, and EHI_074110), 15 START domain homologs other than Sec14 (Figure 2). ORP homologs might be associated with cytoskeleton organization, cell adhesion, and motility, as reported in higher eukaryotes (Goldfinger et al., 2007) and previously discussed in section Cytoskeleton organization, adhesion, and motility Other than ORP, a few START domain homologs could also be associated with cytoskeletal reorganization and migration, as previously reported in a cancer cell line (Alpy and Tomasetto, 2005). Adhesion with the host gut epithelia and migration through the tight junction between host cells are two key pathogenic processes presented by *E. histolytica* trophozoites (Tavares et al., 2005; Franco-Barraza et al., 2006), which also involve the receptor-ligand mediated signaling cascades, in which PIs play indispensable roles.

## Concluding remarks

We have discovered a panel of conserved and lineage-specific LTPs in *E. histolytica* by domain-based survey of LTP homologs in AmoebaDB. The *E. histolytica* genome possess single PRELI domain containing protein (EHI_143630). It is worth investigating whether this PRELI domain containing protein is involved in mitosomal transport. *E. histolytica* possess a single START domain containing protein (EHI_155260) with potential NLS and NES, indicating its potential role as a nuclear lipid transporter and its ability of nucleocytoplasmic shuttling depending upon physiological conditions. Upregulation of gene expression of its two homologs (EIN_257190, EIN_107840) in *E. invadens* during encystation (De Cádiz et al., 2013), indicates its potential role in cell differentiation as well as disease transmission. The *E. histolytica* genome possess a repertoire of START domain containing proteins. Some of them can function as potential PITP similarly as in *Plasmodium* sp. (Van Ooij et al., 2013; Hill et al., 2016). This is also conceivable because phosphoinositides are the essential signaling phospholipids for *E. histolytica*, which depends greatly on receptor-ligand mediated signaling processes. On the other hand, *E. histolytica* lacks canonical PITP homologs from higher eukaryotes. Furthermore, *E. histolytica* lacks any homologs of SMP containing proteins, which are involved in the organization of various MCSs and ERMES complex (Helle et al., 2013). The absence of canonical mitochondria, ER, and Golgi apparatus, in the forms found in higher eukaryotes, could possibly justify this observation. The identified LTP repertoire encompassing ORP, START, SEC14, and PRELI present in *E. histolytica* ensures the complexity and biological significance of LTPs in a variety of cellular processes: lipid transport between cellular compartments, adherence, phagocytosis, trogocytosis, endocytosis, signal transduction, vesicular traffic, cytoskeletal reorganization, nuclear regulation, lipid presentation, and sensing. Further studies are needed to elucidate the role of individual LTPs on the biology and pathogenesis of this parasite. Once *E. histolytica*-specific LTPs and lipid transfer mechanisms are identified, they may potentially provide a novel drug target against this medically important parasite. Since most of existent chemical interventions against lipid signaling pathways target particular lipid metabolizing enzymes (Nile et al., 2014; Khan et al., 2016), such intervention often exerts specific and limited overall effects to the eukaryotic cells, e.g., cancer cells. In addition, parasitic organisms often have highly adaptable nature and a bypass mechanism to overcome the effects caused by various chemical insults, results in the generation of drug resistance. Thus, chemical inhibition of parasite-specific LTPs might be a reasonable solution to the problem because LTPs are involved in a wide range of multiple cellular processes described above.

## Author contributions

All authors listed have made a substantial, direct and intellectual contribution to the work, and approved it for publication.

### Conflict of interest statement

The authors declare that the research was conducted in the absence of any commercial or financial relationships that could be construed as a potential conflict of interest.
